# High-Dose Methotrexate in Pediatric Acute Lymphoblastic Leukemia: Predictors of Delayed Clearance and the Effect of Increased Hydration Rate on Methotrexate Clearance

**DOI:** 10.7759/cureus.8674

**Published:** 2020-06-17

**Authors:** Aaron R Chen, YunZu M Wang, Mark Lin, Dennis J Kuo

**Affiliations:** 1 Division of Biological Sciences, University of California San Diego, San Diego, USA; 2 Bone Marrow Transplant and Immune Deficiency, Cincinnati Children's Hospital Medical Center, Cincinnati, USA; 3 Department of Pharmacy, University of California San Francisco Benioff Children's Hospital, Oakland, USA; 4 Pediatric Hematology/Oncology, University of California San Diego, San Diego, USA

**Keywords:** methotrexate, acute lymphoblastic leukemia, pharmacology

## Abstract

Objectives

High-dose methotrexate (HDMTX) is an important chemotherapeutic agent in the treatment of many cancers. Identification of the predictors of poor clearance during HDMTX infusions could advance the introduction of improved supportive care to prevent toxicities and reduce hospital length of stay. The purpose of this study was to identify relationships between patient physical characteristics and HDMTX clearance in the treatment of pediatric acute lymphoblastic leukemia (ALL). At our hospital, patients who have delayed methotrexate (MTX) clearance during a cycle of HDMTX receive an increased rate of hydration with subsequent cycles. This increase in hydration rate was examined for its potential to mitigate predictors of poor clearance and to prevent nephrotoxicity.

Methods

This study retrospectively examined the treatment records of 87 pediatric patients diagnosed with ALL who were treated on or according to Children’s Oncology Group (COG) protocols AALL0232, AALL0434, AALL1131, and AALL1231. Each patient received four cycles of HDMTX (5 g/m^2^ over 24 hours) at two-week intervals. Patients received either 125 ml/m^2^/hour (standard) or 200 ml/m^2^/hour (delayed clearance protocol) hydration before, with, and after each infusion. MTX levels taken at 24-, 42-, and 48-hour time points were used as an indirect measure of drug clearance. Two-tailed inference for ordinary least squares regression and both heteroskedastic and paired two-tailed t-tests were performed to identify physical characteristics associated with delayed MTX clearance and the effects of hydration rate on MTX clearance, respectively.

Results

Patient age and body surface area (BSA) were found to have statistically significant (p<0.05) positive associations with the serum MTX levels at 24, 42, and 48 hours in cycle 1. Age and BSA were significant only at the 24-hour time point in cycles 2 and 4. Weight alone was not associated with delayed MTX clearance. For patients who had delayed MTX clearance once and thus received the delayed clearance protocol in subsequent cycles, increasing the hydration rate from 125 to 200 ml/m^2^/hour was associated with a statistically significant decrease in average MTX levels as well as serum creatinine levels at the 24-, 42-, and 48-hour time points. Once patients with delayed clearance received the 200 ml/m^2^/hour rate of hydration, the history of prior poor clearance lost its predictive value for serum MTX levels and delayed clearance.

Conclusions

These results suggest that patient age and BSA are significant predictors of MTX clearance if all patients receive the same rate of hydration. Age and BSA affect the distribution phase of MTX kinetics, with downstream effects in the elimination phase. Increased hydration mitigates the effects of these physical characteristics on the elimination phase kinetics by improving renal elimination of MTX, causing a loss of significance of age and BSA as predictors of MTX levels in subsequent cycles at the 42- and 48-hour time points, but with less effect at 24 hours. Thus, hyperhydration regimens prior to cycle 1 of HDMTX could be considered for patients presenting with risk factors of advanced age or high BSA to avoid delayed clearance.

## Introduction

High-dose methotrexate (HDMTX) is an important chemotherapeutic agent in the treatment of many cancers, including acute lymphoblastic leukemia (ALL), non-Hodgkin lymphoma, and osteosarcoma. HDMTX is known to cause multiple side effects, including nephrotoxicity, mucositis, hepatotoxicity, neurotoxicity, and myelosuppression. It is eliminated from the body through the kidneys by both passive glomerular filtration and active tubular reabsorption and secretion [1]. To minimize the side effects, intravenous hydration, leucovorin rescue, and proper monitoring of serum creatinine and methotrexate (MTX) levels are crucial. Some patients may be predisposed to experiencing more side effects than others. Patients with third space fluid collections experience slowed redistribution of MTX from extravascular fluid spaces during the distribution phase of MTX kinetics. Due to decreased renal function, patients with baseline renal impairment suffer from decreased clearance rate during the elimination phase of MTX kinetics [2]. Drug-drug interactions or other nephrotoxic medications have also been known to cause a decrease in MTX clearance as well and are thus avoided in patients receiving HDMTX. 

Hyperhydration regimens have been implemented in many institutions in order to decrease the risk of toxicities associated with HDMTX. With proper hydration, the risk of MTX precipitation in the renal tubules and the resulting nephrotoxicity is decreased. Urinary alkalinization is also utilized, as an increase in urine pH has been shown to increase the renal clearance of MTX [3]. These supportive care measures help decrease the risk of nephrotoxicity as its mechanism is hypothesized to be due to either direct tubular toxicity, pH-dependent precipitation of MTX in the renal tubules, or both [4]. Leucovorin rescue is another common toxicity-management regimen to protect the patient from significant neurotoxicity, gastrointestinal inflammation, and myelosuppression. As leucovorin reactivates dihydrofolate reductase, its administration counteracts the effects of MTX with the intention of protecting normal cells from prolonged exposure to MTX [5]. Leucovorin is thus started later after the completion of the HDMTX administration, so as not to hinder MTX’s tumoricidal effects.

Despite these known factors that influence MTX clearance, some patients still develop nephrotoxicity and display varied clearance rates due to the intra- and interpatient variability of MTX pharmacokinetics. Studies have compared the area under the curve (AUC) of MTX and correlated them with the occurrence of toxicities, but the calculated AUC of MTX is not a practical way to adjust interventions in real-time in most clinical settings [6]. MTX clearance is a vital component of calculating the AUC of MTX as the AUC is the dose divided by the clearance. To gain a better understanding of MTX toxicities, it is crucial to understand all the factors that can influence MTX clearance. Patient physical characteristics may affect HDMTX clearance but this has not been well-established. The purpose of this study was to identify relationships between the patient physical characteristics and HDMTX clearance in the treatment of pediatric ALL. In our study, we gauged MTX clearance by the achievement of specific serum MTX concentrations at given time points as outlined by the administration protocols [7]. While timed serum MTX concentrations are different from MTX clearance rates, the two are correlated, and the former serves as a practical surrogate for the latter. Failures to achieve specified MTX levels in time are clinically used to determine if the patient has prolonged exposure to a toxic concentration of MTX, which may result in renal impairment or other toxicities. As such, our study focused on identifying physical characteristics that predicted delayed MTX clearance, as gauged by timed serum MTX concentrations, and a potential strategy to mitigate their effects. We examined the associations of increased intravenous hydration rate with reduced serum MTX levels as well as reduced creatinine levels.

## Materials and methods

This study retrospectively examined the treatment records of pediatric patients diagnosed with high-risk ALL at a large children’s hospital (Rady Children’s Hospital, San Diego, CA) who were treated on or according to Children’s Oncology Group (COG) protocols AALL0232, AALL0434, AALL1131, and AALL1231 [7]. These patients were selected for review on the basis of the similar HDMTX administration guidelines for these protocols. 

Each patient was scheduled to receive four cycles of HDMTX (5 g/m^2^ over 24 hours) at two-week intervals. The date of the initiation of each cycle could be delayed by factors such as hematologic toxicity, mucositis, hepatotoxicity, nephrotoxicity, and scheduling issues. Each cycle of HDMTX consisted of 500 mg/m^2^ MTX infused intravenously as a bolus over 30 minutes followed by 4500 mg/m^2^ infused over the next 23.5 hours. Intrathecal MTX (8-15 mg depending on the patient’s age) was also administered in cycles 1 and 3, delivered within six hours of the initial HDMTX bolus.

In each cycle, patients initially received intravenous fluids containing 30 mEq NaHCO_3_/L at 125 ml/m^2^/hour as the standard hydration per protocol. Pre-hydration was given for six to eight hours or until the urine specific gravity was ≤1.010 and the urine pH was ≥7.0 and ≤8.0 [8]. Hydration was continued at least until 48 hours after HDMTX infusion was started and until the decreasing serum MTX levels demonstrated appropriate clearance. Appropriate MTX clearance was defined as having a serum MTX level less than 1.0 μM at 42 hours and 0.4 μM at 48 hours after the start of the MTX infusion. Delayed clearance was defined as any elevation of MTX levels beyond the appropriate clearance guidelines. Leucovorin 15 mg/m^2^ was given at hours 42, 48, and 54 after the start of HDMTX, with the dose and/or frequency increased if the clearance was delayed, per protocol [9]. Patients who had delayed MTX clearance with a prior cycle of HDMTX received an increased rate of hydration with similar timing before, with, and after HDMTX infusion at 200 ml/m^2^/hour in subsequent cycles. MTX levels measured at 24-, 42-, and 48-hour time points after each initial bolus of HDMTX were used as a serial parameter for indirectly measuring MTX clearance. In a clinical setting, serum MTX levels at these time points are used to monitor MTX elimination from the body. Serum creatinine levels, also drawn at 24-, 42-, and 48-hour time points were used as a measure of kidney function in the elimination of MTX. These timed MTX and creatinine levels were used to determine the appropriate leucovorin rescue regimen, hydration rate, and if additional supportive measures such as glucarpidase are necessary in cases of delayed clearance.

Creatinine (an endogenous renally eliminated metabolic byproduct) levels in the blood are an indirect measure of the kidney filtering capacity, with higher levels indicating poorer filtering capacity [10]. For the purpose of our analysis, serum creatinine levels drawn after the start of the HDMTX infusion were analyzed as outcome variables to assess the effect of the prescribed intravenous hydration regimens on renal function. The pre-admission serum creatinine levels were not analyzed as independent variables as these levels are very susceptible to variability depending on the individual patient’s oral intake habits before admission. Furthermore, those effects of oral hydration at home on the pre-chemotherapy baseline creatinine would be mitigated by the pre-hydration that all the patients would receive prior to the HDMTX infusion being started. Additionally, if the serum creatinine drawn prior to admission to start HDMTX is ≥1.5 times the prior baseline creatinine, the renal function would be considered impaired and admission would be delayed until creatinine levels return closer to baseline, thereby biasing pre-admission creatinine levels toward normalcy.

Two-tailed inference for ordinary least squares regression was used to determine the significance of associations between the hypothesized predictors [age, body surface area (BSA), and weight] and MTX clearance. This form of linear regression was selected for its simplicity and robustness with continuous independent and dependent variables. Since MTX levels are used as a practical analog for clearance, the model was not intended to predict a patient’s MTX levels at a given time point based on observed physical characteristics. Instead, the selected model was intended to determine the significance of association while avoiding overfitting. Regression models were tested against the null hypothesis that the slope of the model would be zero if there was no association. Given that BSA and weight increase with age, their variance inflation factors were high, indicating a strong correlation between predictors. Thus, regression inference for each variable against MTX levels was conducted independently to prevent the detection of false significance due to multicollinearity. This method does allow more potential for the effects of confounding variables, such as drug interactions or renal impairment. However, as noted in the COG protocols, known drug interactions were avoided during HDMTX administration [9]. Furthermore, baseline renal function was assessed prior to every cycle of HDMTX and administration only carried out if renal impairment was not found [8].

Heteroskedastic two-tailed t-tests were used in interpatient comparisons of clearance to assess the effects of increased pre-hydration rates on MTX clearance as well as creatinine levels for the group as a whole. This model compared the average MTX and creatinine levels across all patients at a given time point with that of patients receiving increased hydration in the next cycle, accounting for differences in sample variance. Paired two-tailed t-tests were used to evaluate intrapatient clearance changes in response to increased pre-hydration rates compared to previous cycles; t-tests on MTX levels and creatinine levels were performed independently of one another. In all tests, a p-value of <0.05 was considered statistically significant.

## Results

Using the ALL protocol criteria described above, 90 patients between the ages of 2-18 years were identified. Three of the 90 patients surveyed did not complete all four cycles and their data from all cycles were excluded from all inter-cycle analyses to prevent influence from these outliers. A summary of the 87 analyzed patients’ physical characteristics is given in Table 1. Of the 348 total cycles of HDMTX that these patients completed, 92 (26.4%) had delayed MTX clearance, with 52 out of 87 patients (59.8%) experiencing at least one cycle of delayed clearance at some point during treatment. In the first cycle of HDMTX administration, 25 of the sampled patients had delayed clearance.

**Table 1:** Patient physical characteristics* *Summary of physical characteristics of patients whose medical records were analyzed (n=87) BSA: body surface area [calculated as height (meters) squared]; BMI: body mass index [calculated as weight (kilograms) divided by height (meters) squared]; SD: standard deviation

Variable	Average ± SD	First quartile	Median	Third quartile	Range
Age (years)	9.40 ± 5.00	4.32	9.70	14.10	2.01-18.02
Weight (pounds)	77.90 ± 46.37	37.34	62.23	107.47	22.49-198.63
BSA (m^2^)	1.12 ± 0.45	0.72	1.04	1.49	0.48-2.13
BMI (kg/m^2^)	18.19 ± 4.48	14.68	16.84	20.16	12.83-31.13
Gender	46 males, 41 females	-	-	-	-

Predictors of delayed clearance

Patient age and BSA were found to have statistically significant (p<0.05) positive linear associations with the serum MTX levels at 24, 42, and 48 hours in cycle 1. Age and BSA were significant only at the 24-hour time point in cycles 2 and 4. Weight alone was associated with higher 24-hour MTX levels in cycles 2 and 4, but this finding was likely due to some residual multicollinearity with age and BSA. Overall, the weight alone was not associated with MTX clearance.

**Table 2:** Ordinary least squares inference p-values for physical characteristics associated with serum MTX levels Serum MTX levels were drawn at 24, 42, and 48 hours after initial infusion. A p-value of <0.05 was considered statistically significant. Slopes for all significant associations were positive MTX: methotrexate; BSA: body surface area

Time point and cycle	Weight	Age	BSA
Cycle 1: 24-hour MTX	0.065	0.035	0.042
Cycle 1: 42-hour MTX	0.201	0.027	0.017
Cycle 1: 48-hour MTX	0.217	0.032	0.010
Cycle 2: 24-hour MTX	0.006	0.001	0.004
Cycle 2: 42-hour MTX	0.928	0.743	0.841
Cycle 2: 48-hour MTX	0.662	0.143	0.366
Cycle 3: 24-hour MTX	0.393	0.393	0.390
Cycle 3: 42-hour MTX	0.153	0.221	0.188
Cycle 3; 48-hour MTX	0.749	0.923	0.919
Cycle 4: 24-hour MTX	0.016	0.009	0.010
Cycle 4: 42-hour MTX	0.389	0.901	0.562
Cycle 4: 48-hour MTX	0.580	0.388	0.532

Effect of increasing hydration

**Figure 1:**
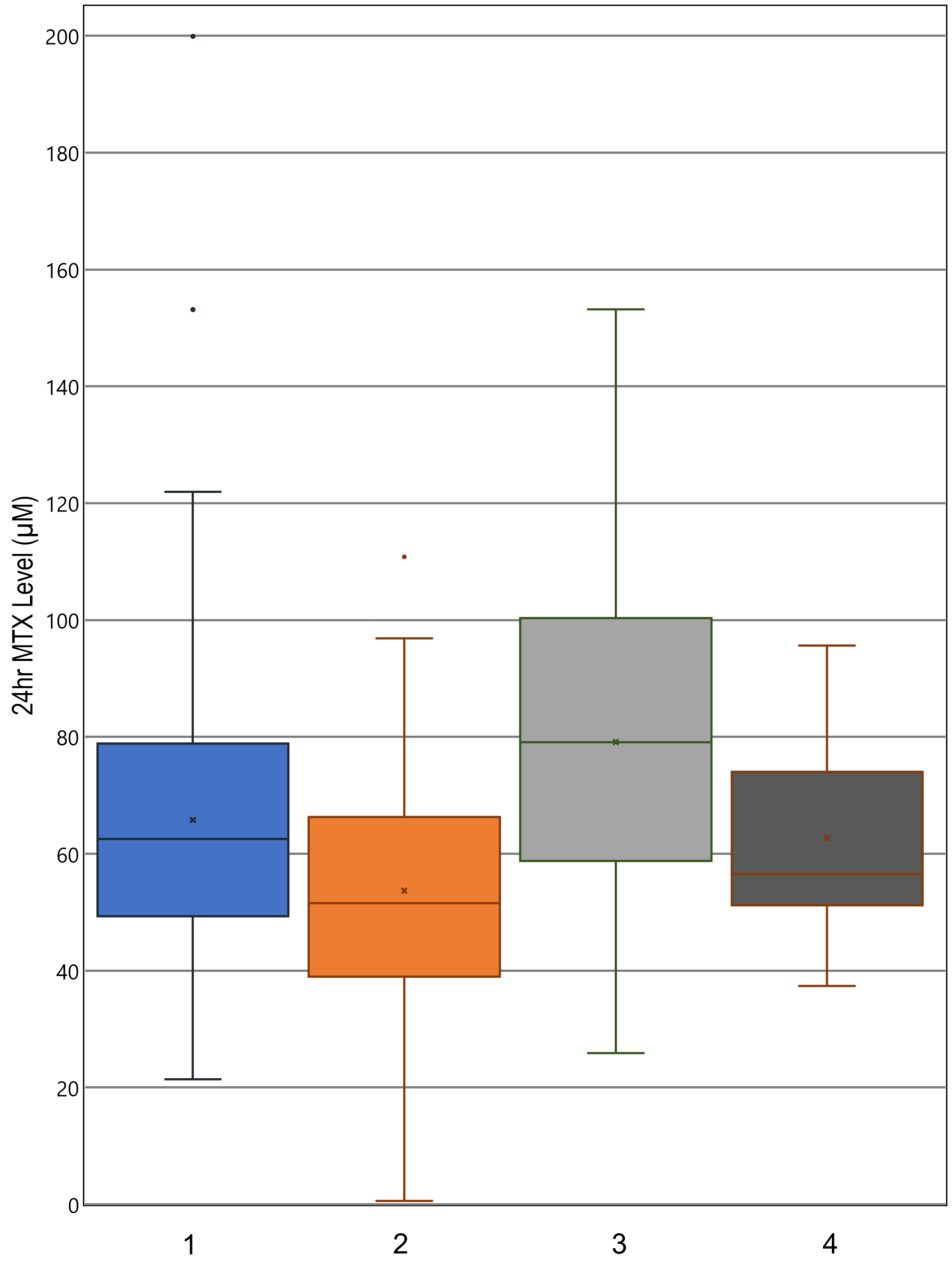
24-hour serum MTX level distributions Boxplots indicate median, range, and quartiles. X marks the mean; 1: cycle 1 levels, all patients; 2: cycle 2 levels, all patients; 3: cycle 1 levels, patients delayed in cycle 1; 4: cycle 2 levels, patients delayed in cycle 1 MTX: methotrexate

**Figure 2:**
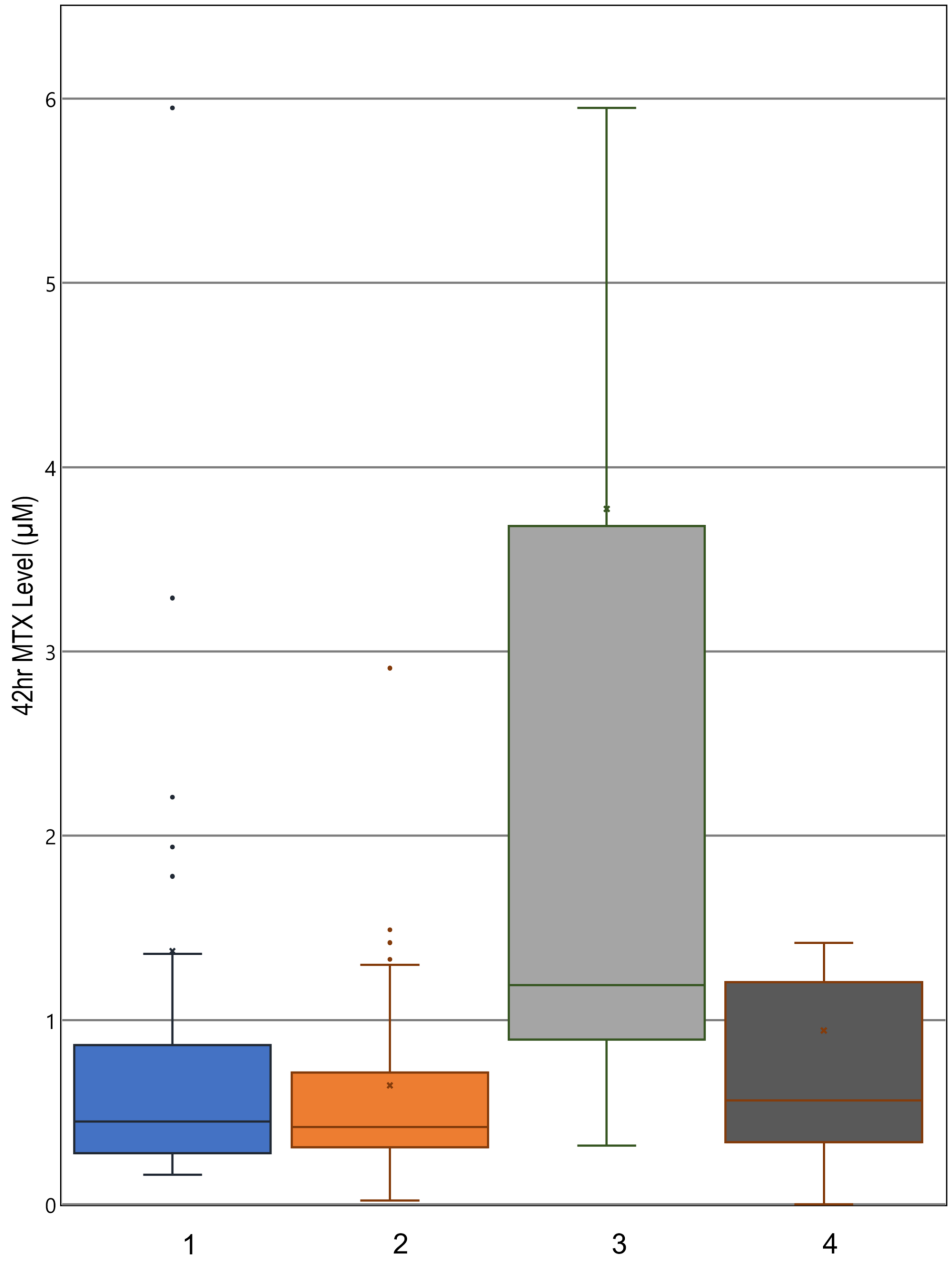
42-hour serum MTX level distributions Boxplots indicate median, range, and quartiles. X marks the mean; 1: cycle 1 levels, all patients; 2: cycle 2 levels, all patients; 3: cycle 1 levels, patients delayed in cycle 1; 4: cycle 2 levels, patients delayed in cycle 1 MTX: methotrexate

**Figure 3:**
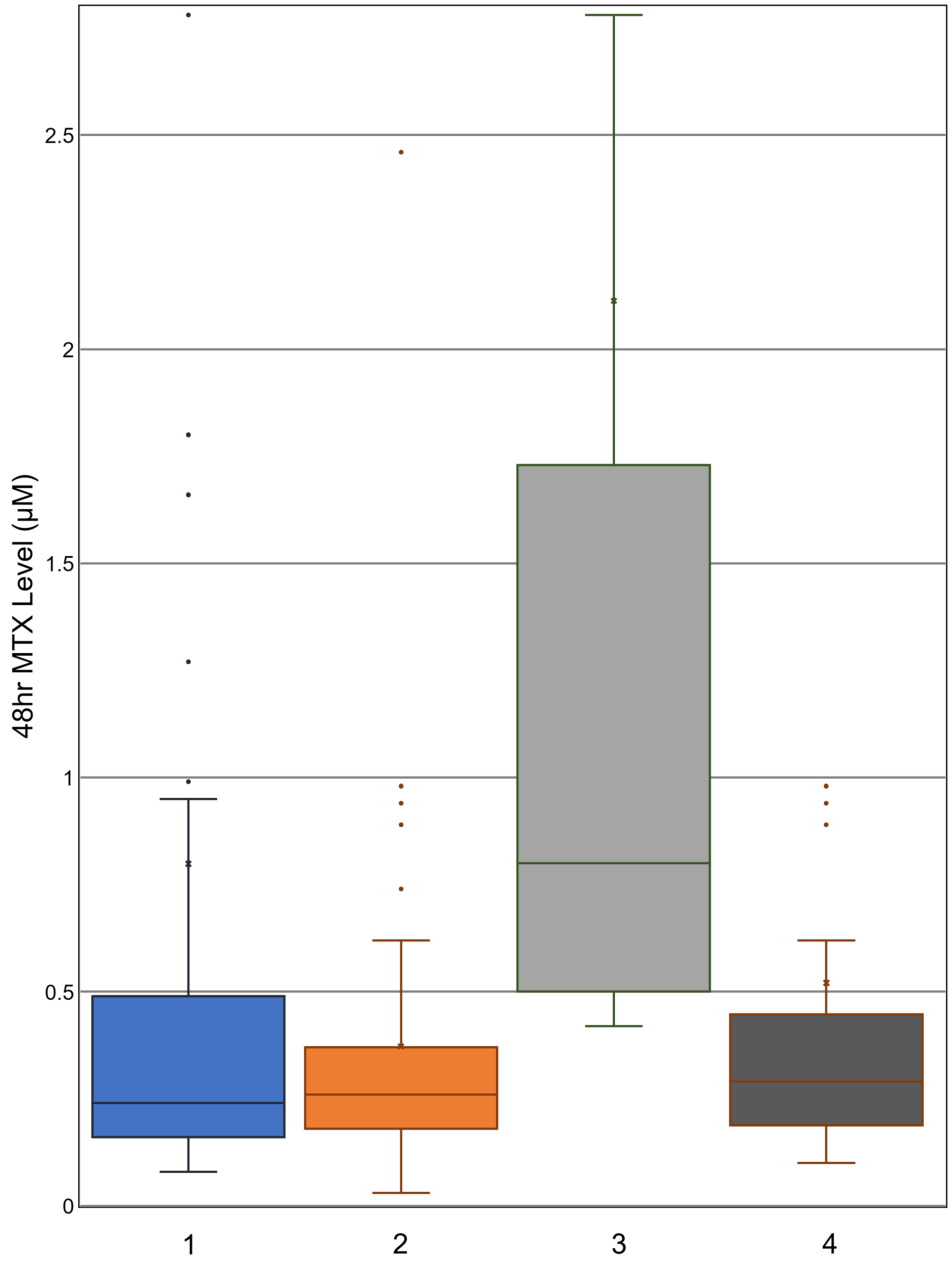
48-hour serum methotrexate level distributions Boxplots indicate median, range, and quartiles. X marks the mean; 1: cycle 1 levels, all patients; 2: cycle 2 levels, all patients; 3: cycle 1 levels, patients delayed in cycle 1; 4: cycle 2 levels, patients delayed in cycle 1 MTX: methotrexate

Patients who had delayed MTX clearance during any cycle received the delayed clearance protocol in subsequent cycles, where the hydration rate was increased from 125 to 200 ml/m^2^/hour. The beneficial effect of this change is shown in Figure 1. Comparison of columns 1 and 2 shows that the mean, median, and ranges for the 24-hour MTX levels decreased between cycle 1 and cycle 2 for the entire sampled patient population. Improvement in serum MTX of patients delayed in cycle 1 and receiving the increased hydration in cycle 2 explains the significantly decreased average serum MTX of the whole population. Comparing columns 3 and 4 of Figure 1 supports this notion, as it demonstrates a significant decrease in 24-hour serum MTX for the patients with delayed clearance in cycle 1 compared to their cycle 2 levels, with the clinical difference being the increased hydration given in cycle 2. In this comparison of columns 3 and 4, from cycle 1 to cycle 2, the range of MTX levels tightens along with decreases in the median value, the 25th percentile value, and the 75th percentile value. The median 24-hour serum MTX decreased by 19.82 μM, with a p-value of 0.0270. This would indicate a decrease in serum MTX in a majority of patients, rather than a decrease in the mean due to selective large decreases in the population’s outliers. Serum MTX level measurements taken at 42- and 48-hour time points show similar results (Figures 2, 3). These decreases in average MTX levels for all patients at the 24-, 42-, and 48-hour time points from cycle 1 to cycle 2 were found to be statistically significant (Table 3, column A).

**Table 3:** Increased hydration serum MTX level t-test p-values A and B are paired two-tailed t-tests while C is a heteroskedastic t-test. (1) cycle 1 levels, all patients. (2) cycle 2 levels, all patients. (3) cycle 1 levels, patients delayed in cycle 1. (4) cycle 2 levels, patients delayed in cycle 1. A p-value of <0.05 was considered statistically significant MTX: methotrexate

	A: (1) vs. (2)	B: (3) vs. (4)	C: (1) vs. (4)
24-hour MTX	0.0008	0.0270	0.6572
42-hour MTX	0.0310	0.0133	0.4989
48-hour MTX	0.0318	0.0084	0.5212

There was also a statistically significant decrease in the cycle 2 average 24-, 42-, and 48-hour MTX levels for patients with delayed clearance in cycle 1 (Table 3, column B). The new distribution of MTX levels for these patients with a history of delayed clearance was not significantly different from that of all patients receiving HDMTX for the first time (Table 3, column C). As these patients with a history of delayed clearance demonstrated improvement, the overall decrease in average MTX levels to that of the initial general population is explained by the improved clearance of the patients who had delayed clearance in the previous cycle. Thus, once patients with delayed clearance received the 200 ml/m^2^/hour rate of hydration in the subsequent cycles, the history of prior poor clearance lost its predictive value for timed serum MTX levels and delayed clearance (Table 3). When the patients were categorized by hydration rate, age and BSA were no longer significant predictors of clearance in cycles 2, 3, and 4 (Table 4). This would indicate that increased hydration rate diminishes the effect of these physical predictors as well.

**Table 4:** Regression inference p-values for physical characteristics associated with serum MTX levels categorized by hydration rate* *Includes all patients in cycle 2-4 levels at 42-hour and 48-hour time points MTX: methotrexate; BSA: body surface area

		Cycle 2		Cycle 3		Cycle 4	
		125 ml/m^2^/hour	200 ml/m^2^/hour	125 ml/m^2^/hour	200 ml/m^2^/hour	125 ml/m^2^/hour	200 ml/m^2^/hour
Weight	42 hours	0.915	0.822	0.387	0.250	0.351	0.690
	48 hours	0.824	0.776	0.851	0.710	0.725	0.344
Age	42 hours	0.488	0.643	0.178	0.745	0.829	0.957
	48 hours	0.915	0.284	0.643	0.956	0.989	0.220
BSA	42 hours	0.828	0.955	0.385	0.328	0.413	0.889
	48 hours	0.970	0.559	0.961	0.807	0.701	0.249

Similar results are observed with serum creatinine levels drawn from patients at the same 24-, 42-, and 48-hour time points. There was a statistically significant decrease in average serum creatinine of the patients between cycles 1 and 2 (Table 5, column A), with again the clinical difference between cycles being that patients with a history of delayed clearance in cycle 1 received the increased hydration regimen in cycle 2. Similarly to the findings with serum MTX levels, patients delayed in cycle 1 demonstrated significantly reduced cycle 2 serum creatinine levels, with much greater decreases per patient than the whole population (Table 5, column B). When the cycle 2 serum creatinine levels of patients delayed in cycle 1 were compared to the levels of all patients in cycle one, there was little difference in average serum creatinine level (Table 5, column C). Their new distribution of creatinine levels was found to be not significantly different from that of a random group of first-time HDMTX recipients, suggesting that increased hydration may mitigate some of the nephrotoxic effects of MTX.

**Table 5:** Serum creatinine t-test p-values with increased hydration rate A and B are paired two-tailed t-tests while C is a heteroskedastic t-test. Avg Δ is the average change in serum creatinine of patients in the indicated analyses. (1) cycle 1 levels, all patients. (2) cycle 2 levels, all patients. (3) cycle 1 levels, patients delayed in cycle 1. (4) cycle 2 levels, patients delayed in cycle 1. A p-value of <0.05 was considered statistically significant

	A: Avg Δ	A: (1) vs. (2)	B: Avg Δ	B: (3) vs. (4)	C: Avg Δ	C: (1) vs. (4)
24-hour creatinine	-0.041 mg/dL	0.0054	-0.100 mg/dL	0.0047	-0.010 mg/dL	0.9297
42-hour creatinine	-0.029 mg/dL	0.0794	-0.101 mg/dL	0.0118	-0.020 mg/dL	0.6297
48-hour creatinine	-0.031 mg/dL	0.0636	-0.109 mg/dL	0.0086	-0.008 mg/dL	0.9359

Inter-cycle creatinine levels drawn at 24 hours were also compared for patients who did not receive the same rate of hydration in consecutive cycles (Table 6). This considered patients who were not delayed in cycle 1 and again received 125 ml/m^2^/hour hydration in cycle 2, as well as patients delayed in cycle 1 who received 200 ml/m^2^/hour in cycles 2 and 3. In both instances, regardless of whether patients consistently received 125 ml/m^2^/hour or 200 ml/m^2^/hour on the two consecutive cycles, there was no significant increase nor decrease in creatinine levels between the two cycles at the 24-hour time point. Therefore the observed average decrease in creatinine in patients who initially received 125 ml/m^2^/hour and then switched in a subsequent cycle to 200 ml/m^2^/hour was likely attributable to the increased hydration rate. It is thus indicated that the increased hydration may help improve glomerular filtration.

**Table 6:** 24-hour serum creatinine of patients receiving fixed hydration across cycles Columns A and B represent patients not delayed in cycle 1. Columns C and D represent patients delayed in cycle 1. These groups are mutually exclusive. P-values are from paired two-tailed t-tests of A/B and C/D. A p-value of <0.05 was considered statistically significant SD: standard deviation

	A: Cycle 1 125 ml/m^2^/hour	B: Cycle 2 125 ml/m^2^/hour	C: Cycle 2 200 ml/m^2^/hour	D: Cycle 3 200 ml/m^2^/hour
Mean ± SD	0.368 ± 0.137 mg/dL	0.352 ± 0.124 mg/dL	0.412 ± 0.109 mg/dL	0.401 ± 0.087 mg/dL
P-values	0.3359		0.4183	

## Discussion

For patients receiving the same rate of hydration, patient age and BSA are significantly associated with MTX clearance, with older and larger patients having a predisposition for slower clearance. In cycle 1, 28.7% of sampled patients experienced delayed clearance, roughly consistent with the 30.6% risk for delayed clearance for the first-time receiving of HDMTX documented in another study [11]. Increasing the rate of NaHCO_3_ containing intravenous hydration fluids increases the urine alkalization and the glomerular filtration rate, improving the solubility of MTX for renal elimination. In our study, this mitigated the effects of these physical characteristics (age and BSA) on the elimination phase kinetics, causing their loss of significance as predictors of MTX levels in subsequent cycles at the 42- and 48-hour time points. There is less of a beneficial effect on the 24-hour MTX levels since those earlier levels are associated with distribution phase kinetics. Patient age and BSA are directly correlated with the volume of distribution and heavily influence this phase of MTX kinetics, with downstream effects in the elimination phase. Renal function, critical to the elimination of MTX from the body, was demonstrated to be improved with increased hydration, indicated by reduced serum creatinine. Thus, it appears that the mechanism by which increased hydration rates reduce serum MTX levels is via acceleration of its elimination phase kinetics.

MTX is ubiquitous in pediatric oncology chemotherapy. Due to its well-known toxicities, there are multiple algorithmic flowcharts detailing the contingencies of HDMTX administration, defining fluid rate changes, serum level monitoring, leucovorin rescue dosage and timing, and criteria to administer emergency glucarpidase when the above measures have failed to adequately protect the patient from what will be excessive toxicity [12].

While it is second nature to pediatricians to order medications using weight-based dosing, oncology adds an additional level of complexity with BSA-based dosing. Standard guidelines for both HDMTX and its accompanying intravenous fluids are based on BSA. While other known intrinsic risk factors for poor MTX clearance and MTX toxicity, such as age and gender, are not mathematically accounted for, BSA is the single variable in calculating the starting intravenous fluid rate and the dose of MTX [13]. In our analysis, despite the MTX dose and intravenous fluid rate being calculated according to the BSA, BSA, along with age, remains a significant predictor of elevated MTX levels at 24 hours in 3 of 4 cycles of HDMTX. In cycles 2-4, when patients with delayed MTX clearance during a prior cycle are administered increased fluid hydration rates throughout the HDMTX infusion, the significance of age and BSA disappears when patients are analyzed in subgroups according to their fluid rate, indicating that a higher fluid rate mitigates the effects of age and BSA. This contrasts with cycle 1 when all patients are started on fluids at 125 ml/m^2^/hour, and age and BSA are significant predictors of elevated serum MTX levels at all (24, 42, and 48) hours.

Additional risk factors for delayed MTX clearance described in earlier studies, such as male gender, low urine pH, emesis during infusion, prior renal injury, or known third spacing, should also suggest a need for a higher fluid rate [4,13,14]. These rates were established after papers, such as that of Relling et al. in 1994, demonstrated that increasing hydration reduced the frequency of high-risk MTX concentrations and toxicities [14]. Modifications such as extended pre-hydration time prior to HDMTX infusion have not been shown to affect MTX levels or toxicity [15,16]. The reduction of serum creatinine by increased hydration rate opens the potential of investigating its nephroprotective effects. HDMTX is thought to be increasingly nephrotoxic in subsequent cycles and administering hydration at higher rates in those cycles may mitigate these effects. Although the need for hydration and alkalinization was demonstrated several decades ago, it remains a delicate balance between the therapeutic requirement of systemic exposure to a high MTX concentration, the toxicities of delayed clearance, and the practical concerns of administering supraphysiologic rates of intravenous hydration, such as electrolyte abnormalities and very frequent urination [14,17-20].

The findings of Kinoshita et al. established that higher sodium concentration in hydration improves MTX clearance and suggest that increased amounts of sodium in the body improve MTX elimination by means of improved MTX solubility [20]. Increased hydration rate may be a means to achieve the same end using a lower concentration of sodium. Thus, a balance may potentially be found by increasing both the sodium concentration and the intravenous fluid rate that avoids the concerns of electrolyte imbalance or excessive fluid intake.

Our study is limited by its small size; moreover, it represents the experience from a single institution only. For these reasons, we sought to identify, rather than statistically model, significant predictors of a predisposition to poorer MTX clearance and to examine whether increased intravenous hydration rate could mitigate those effects. Given the findings of our study, we would suggest that pediatric oncology providers could consider increased age and BSA as risk factors for delayed MTX clearance after HDMTX infusions. Furthermore, this study demonstrated that increasing the hydration rate to 200 ml/m^2^/hour decreased the effect of age and BSA on the rate of MTX clearance. Thus, in selected patients, particularly those with other factors that would predict delayed MTX clearance, starting at increased hydration rates in the first cycle the patients receive HDMTX may reduce the incidence of poor MTX clearance and toxicity.

## Conclusions

Patient age and BSA appear to be significant predictors of MTX clearance if all patients receive the same rate of hydration. Age and BSA are hypothesized to affect the distribution phase of MTX kinetics, with downstream effects in the elimination phase. Increased hydration potentially mitigates the effects of these physical characteristics on the elimination phase kinetics by improving glomerular filtration and thus renal elimination of MTX, causing age and BSA to lose significance as predictors of MTX levels in subsequent cycles at the 42- and 48-hour time points, but with less effect at 24 hours. Hyperhydration prior to cycle 1 of HDMTX could thus be considered for patients with higher age or BSA to avoid delayed clearance. Further studies on patient physical characteristics could potentially develop a predictive model that would establish thresholds for each characteristic at which increased risk of delayed clearance warrants additional precautionary measures.
